# Linking academic stress to loneliness, anxiety, and depression: The association of problematic micro-drama use among Chinese university students

**DOI:** 10.1371/journal.pone.0361597

**Published:** 2026-10-27

**Authors:** Haoyuan Yu, Zhibiao Yang

**Affiliations:** Department of Journalism, School of Humanities, Hunan University of Science and Technology, Xiangtan, Hunan, China; Shahid Beheshti University of Medical Sciences, IRAN, ISLAMIC REPUBLIC OF

## Abstract

**Objective:**

Problematic micro-drama use (PMDU) has received increasing attention as micro-dramas have become widely consumed among young adults in China. This study examined the associations between academic stress and PMDU among Chinese university students, explored which dimensions of academic stress were uniquely associated with PMDU, and examined the associations between PMDU and loneliness, anxiety symptoms, and depressive symptoms.

**Methods:**

A cross-sectional online survey was conducted among 510 Chinese university students. Participants completed measures of academic stress, PMDU, loneliness, anxiety symptoms, and depressive symptoms. Multiple linear regression analyses were conducted while controlling for gender and age. The Benjamini–Hochberg false discovery rate (FDR) procedure was applied to relevant families of multiple tests.

**Results:**

Higher academic stress was associated with greater PMDU (β = .447, p < .001). Among the five dimensions of academic stress, learning effectiveness pressure remained significantly associated with PMDU after correction for multiple comparisons (β = .292, FDR-adjusted p < .001), whereas the other dimensions did not show statistically significant unique associations after correction. PMDU was positively associated with loneliness (β = .331), depressive symptoms (β = .323), and anxiety symptoms (β = .305), and all three associations remained significant after FDR correction (all FDR-adjusted p < .001).

**Conclusions:**

Academic stress, particularly stress related to perceived learning effectiveness, was associated with greater problematic micro-drama use among Chinese university students. Higher PMDU was also associated with greater loneliness, depressive symptoms, and anxiety symptoms. These findings identify PMDU as a media-use behavior relevant to both academic stress and psychological well-being, although the cross-sectional design does not establish the direction of these associations.

## 1 Introduction

In recent years, micro-dramas (micro-series) have rapidly emerged as a prominent form of mobile audiovisual entertainment within algorithm-driven video ecosystems [1,2]. Compared with traditional television dramas and long-form video content, micro-dramas are typically characterized by shorter episodes, accelerated narrative pacing, highly condensed plots, and emotionally engaging structures such as cliffhanger endings and strong narrative “hooks” [2,3]. These features are particularly compatible with fragmented viewing contexts and mobile-first media consumption habits. The combination of brief duration and narrative continuity also allows users to obtain immersive entertainment and emotional distraction within relatively short periods of time. At the same time, platform mechanisms such as autoplay, endless feeds, and personalized recommendations further facilitate continuous viewing and prolonged engagement [1,4]. Although micro-dramas may provide convenient entertainment and temporary stress relief, repeated and difficult-to-control viewing may gradually develop into problematic micro-drama use (PMDU), particularly among university students who are highly engaged with mobile media. In the present study, PMDU refers to a pattern of excessive or difficult-to-control micro-drama viewing accompanied by functional interference or negative consequences, including difficulties in controlling viewing, interference with academic activities and attention, and problems in interpersonal interactions. PMDU is conceptualized here as a dimensional pattern of problematic media use rather than a formally recognized clinical disorder.

Against this backdrop, mental health problems among university students have become a pressing issue in higher education, with loneliness, anxiety, and depressive symptoms frequently reported [5]. These concerns may be particularly salient in China, where academic environments can be highly competitive and students often face substantial expectations regarding academic performance and future employment [6,7]. Academic stress is widely recognized as an important correlate of psychological distress [8,9]. However, in contemporary digital environments, the behavioral processes through which academic stress becomes linked to mental health problems remain insufficiently understood [10,11]. Given students’ high exposure to mobile media, one plausible pathway is that academic stress may be associated with greater reliance on micro-dramas as a coping tool, which may in turn be linked to problematic viewing patterns and related psychological difficulties.

Stress and coping perspectives distinguish between problem-focused coping, which aims to modify or resolve the source of stress, and emotion-focused coping, which aims to regulate the emotional responses elicited by stress [12]. In the context of academic stress, micro-drama viewing may be more readily understood as an emotion-focused coping behavior. Rather than directly addressing academic demands, students may turn to micro-dramas to temporarily reduce tension, distract themselves from academic concerns, or regulate negative affect [13–15]. Micro-dramas may be particularly suited to this function because they combine low time commitment with relatively high narrative and emotional engagement. Compared with conventional television dramas and long-form video content, their short episodes can be consumed during brief intervals between academic activities, while their serialized narratives, recurring characters, and cliffhanger structures provide greater narrative continuity than many fragmented short videos [2,3,16]. Thus, micro-dramas may offer students a readily accessible form of temporary emotional relief without requiring the time commitment associated with long-form entertainment.

This coping process may also be understood through the lens of basic psychological needs. Academic difficulties and perceived inefficacy may frustrate students’ need for competence, social disconnection may undermine relatedness, and highly structured academic demands may constrain perceived autonomy [17]. Micro-drama viewing may provide short-term compensatory experiences for these frustrated needs: engaging narratives may offer immediate feelings of reward or vicarious accomplishment, recurring characters and socially shared content may foster a sense of connection, and self-directed viewing allows students to exercise choice over what and when they watch. From a compensatory media use perspective, such experiences may make micro-dramas particularly appealing when psychological needs are frustrated in academic life [13,18]. However, because these psychological needs were not directly measured in the present study, they are considered theoretically plausible mechanisms rather than empirically tested mediators.

Although micro-drama viewing may provide temporary emotional relief, repeated reliance on this form of coping may become problematic when viewing becomes habitual or difficult to control. When micro-drama viewing becomes a primary or habitual activity, it may gradually shift from purposeful relaxation toward compulsive behavior [19,20]. This tendency may be reinforced by platform design features that facilitate binge-like consumption, including continuous autoplay and algorithmic recommendations [21], as well as narrative structures such as strong hooks and cliffhangers that encourage continued viewing [22]. Accordingly, higher academic stress may be associated with greater susceptibility to PMDU.

PMDU may also be associated with multiple domains of mental health problems. Excessive micro-drama viewing may displace sleep [21,22], physical activity [23,24], and academic routines [25,26], thereby increasing fatigue and undermining emotion-regulation capacity [27,28]. It may also reduce time and motivation for offline social interactions, potentially intensifying loneliness [29]. Moreover, when students experience a growing discrepancy between intended and actual viewing (e.g., planning to watch briefly but continuing for hours), negative self-evaluations, perceived loss of control, and guilt may emerge, which can exacerbate anxiety and depressive symptoms [29–31]. Taken together, PMDU is expected to be positively associated with loneliness, anxiety, and depression among university students.

Although research on problematic smartphone use and problematic social media use has expanded rapidly, focused investigation of PMDU remains warranted, particularly in the Chinese university context. Micro-dramas represent a distinctive, narrative-driven form of mobile entertainment designed for rapid consumption and sustained engagement [32], and thus may foster compulsive viewing tendencies through mechanisms not fully captured by broader constructs such as overall screen time or generalized social media use. Moreover, existing scholarship on media-related addictive behaviors has often emphasized either antecedents (e.g., stress) [33] or outcomes (e.g., mental health) [34], while offering less clarity on how specific academic stressors translate into specific psychological difficulties in relation to problematic viewing patterns. Finally, because academic stress is inherently multidimensional [35], identifying which facets of academic stress are most closely tied to PMDU can provide a more actionable understanding than treating stress as a single undifferentiated experience.

Building on these considerations, this study addresses three research questions: (1) whether academic stress is associated with PMDU; (2) which dimensions of academic stress are uniquely associated with PMDU after accounting for their shared variance; and (3) whether PMDU is associated with mental health problems, specifically loneliness, anxiety symptoms, and depressive symptoms. The first and third research questions were hypothesis-driven, whereas the second was exploratory. Given the limited research on PMDU and the lack of sufficient theoretical or empirical evidence to predict which specific dimension of academic stress would show a unique association with PMDU, no directional hypothesis was proposed for RQ2.

The significance of addressing these questions is threefold. Theoretically, the study extends stress-and-coping and compensatory media use perspectives to an emerging and under-theorized media format, positioning PMDU as a behavioral correlate associated with both academic stress and psychological distress. Empirically, it provides evidence from a population at elevated risk for both academic strain and intensive mobile media consumption, helping to examine associations with multiple psychosocial outcomes rather than a single mental health indicator. Practically, the findings may inform university mental health services and digital well-being initiatives by identifying academic stressors relevant to prevention and by suggesting that interventions targeting maladaptive micro-drama use may complement existing stress-management and counseling programs.

Based on the above rationale, the following hypotheses were proposed:

H1: Academic stress is positively associated with problematic micro-drama use among Chinese university students.

H2: Problematic micro-drama use is positively associated with loneliness.

H3: Problematic micro-drama use is positively associated with anxiety symptoms.

H4: Problematic micro-drama use is positively associated with depressive symptoms.

## 2 Methods

### 2.1 Study design, participants, and data collection

This study adopted a cross-sectional survey design to examine the associations among academic stress, problematic micro-drama use, and mental health indicators (loneliness, anxiety symptoms, and depressive symptoms) among Chinese university students. Data were collected through Wenjuanxing (问卷星), a widely used online survey platform in China, between December 5 and December 20, 2025. The questionnaire was distributed using a convenience sampling approach through online student networks and social media platforms. Participation was voluntary, anonymous, and completed electronically after informed consent was obtained.

To be eligible for participation, respondents were required to (1) be currently enrolled as university students in China, (2) be at least 18 years old, and (3) have experience watching micro-dramas. Questionnaires were excluded if they were incomplete, contained obvious response inconsistencies, or showed excessively short completion times indicative of inattentive responding.

A total of 534 questionnaires were initially collected. After data screening and removal of invalid responses, 510 valid questionnaires were retained for the final analyses. The final sample size was considered adequate for the planned regression analyses and exceeded commonly recommended thresholds for multivariable statistical modeling.

### 2.2 Measures

Problematic micro-drama use: Given the absence of established clinical diagnostic criteria for problematic micro-drama use, PMDU was conceptualized in the present study as a dimensional pattern of excessive or difficult-to-control micro-drama viewing accompanied by functional interference or negative consequences, rather than as a formally recognized clinical disorder. PMDU was assessed using a 15-item measure adapted from the Short Video Addiction Scale by Ye et al. [36]. The original scale comprises five dimensions: social communication difficulties (4 items), academic procrastination (3 items), attention concentration difficulties (3 items), interpersonal strain (3 items), and impaired control over short-video use (2 items). For the present study, the items were adapted to the micro-drama context by replacing references to short-video viewing with micro-drama viewing while retaining the substantive meaning and dimensional structure of the original items. Accordingly, the adapted measure captures difficulties in controlling viewing as well as academic, attentional, interpersonal, and social interference associated with micro-drama use. Withdrawal symptoms were not directly assessed by this measure.

All items were rated on a 5-point Likert scale ranging from 1 (Strongly Disagree) to 5 (Strongly Agree). The 15 item scores were summed to generate an overall PMDU score ranging from 15 to 75, with higher scores indicating greater levels of problematic micro-drama use. Because the present study focused on overall PMDU rather than dimension-specific associations, the total score was used in the primary analyses. The factorial structure and additional psychometric properties of the adapted measure were evaluated in the present sample as described below.

Academic Stress: Academic stress was measured using a 24-item scale adapted by Han Dong [37], based on the College Students’ Academic Stress Perception Questionnaire [38]. The scale comprises five dimensions: coursework burden, academic climate, learning effectiveness, academic competition, and employment prospects. All items were rated on a 5-point Likert scale ranging from 1 (Strongly Disagree) to 5 (Strongly Agree). Item scores were summed to calculate the overall academic stress score, with higher scores indicating greater academic stress. Dimension scores were calculated by summing the items belonging to each respective dimension, with higher scores indicating greater stress in that domain.

Loneliness: Loneliness was measured using the 6-item De Jong Gierveld Loneliness Scale (DJGLS) [39]. The Chinese version followed Zhong et al. [40]. All items were rated on a 5-point Likert scale (1 = strongly disagree to 5 = strongly agree). The total loneliness score was calculated by adding the six items, with higher scores indicating greater loneliness.

Anxiety and Depression: Anxiety and depressive symptoms were assessed using the Hospital Anxiety and Depression Scale (HADS) [41]. The HADS consists of 14 items, including two subscales: HADS-Anxiety (HADS-A; 7 items) and HADS-Depression (HADS-D; 7 items). The Chinese (Mandarin) version translated and tested by Li et al. was used [42]. Each item is scored from 0 to 3; subscale scores were computed by summing the relevant items, with higher scores indicating more severe anxiety or depressive symptoms.

### 2.3 Reliability and validity

Internal consistency reliability was assessed using Cronbach’s alpha. Table 1 summarizes the internal consistency reliability and factor-analytic suitability of all study measures as part of the reliability and validity assessment. All measures demonstrated good to excellent internal consistency: PMDU (15 items; *α* = .922), academic stress (24 items; *α* = .951), loneliness (6 items; *α* = .914), depression (7 items; *α* = .888), and anxiety (7 items; *α* = .901). For the adapted PMDU measure, corrected item-total correlations and inter-item correlations were additionally examined to assess item-level consistency.

**Table 1:** Reliability and validity assessment of the study measures.

Scale	No. of items	Cronbach’s *α*	KMO	Bartlett’s test *p*
PMDU	15	0.922	0.914	<.001
AS	24	0.951	0.945	<.001
Loneliness	6	0.914	0.869	<.001
Depression	7	0.888	0.909	<.001
Anxiety	7	0.901	0.909	<.001

PMDU = problematic micro-drama use; AS = academic stress.

The Kaiser–Meyer–Olkin (KMO) measure and Bartlett’s test of sphericity were used to assess the suitability of the item sets for factor analysis. KMO values ranged from.869 to.945 (PMDU:.914; academic stress:.945; loneliness:.869; depression:.909; anxiety:.909), and Bartlett’s tests were significant for all measures (*p* < .001), indicating adequate inter-item correlations for factor analysis (Table 1).

Given the multidimensional structure of the source instrument, confirmatory factor analysis (CFA) was conducted using IBM SPSS AMOS (Version 29.0) with maximum likelihood (ML) estimation to evaluate the factorial validity of the adapted PMDU measure. Three alternative measurement models were examined: (1) a single-factor model in which all 15 items loaded on one general factor; (2) a correlated five-factor model corresponding to the structure of the source instrument; and (3) a second-order model in which the five first-order factors loaded on an overarching PMDU factor. Model fit was evaluated using the chi-square statistic (*χ*^2^), comparative fit index (CFI), Tucker–Lewis index (TLI), root mean square error of approximation (RMSEA) with its 90% confidence interval, and standardized root mean square residual (SRMR). Composite reliability (CR) and average variance extracted (AVE) were additionally calculated for the five first-order factors. Discriminant validity was assessed using the Fornell–Larcker criterion and the heterotrait–monotrait ratio of correlations (HTMT).

### 2.4 Data analysis

Statistical analyses were conducted using IBM SPSS Statistics (Version 29.0), while confirmatory factor analysis was performed using IBM SPSS AMOS (Version 29.0) with maximum likelihood estimation. Descriptive statistics, including means, standard deviations, ranges, skewness, and kurtosis, were calculated for the main study variables. Absolute skewness values below 2 and absolute kurtosis values below 7 were considered acceptable for parametric analyses [43].

Pearson correlations were used to examine associations among PMDU, academic stress, loneliness, anxiety symptoms, and depressive symptoms, as well as among the five dimensions of academic stress. Multiple linear regression analyses were then conducted to examine the association between overall academic stress and PMDU, the unique associations of the five academic stress dimensions with PMDU, and the associations of PMDU with loneliness, anxiety symptoms, and depressive symptoms. Gender and age were included as covariates in all regression models.

Regression assumptions were assessed by examining residual distributions, variance inflation factor (VIF) values, the Breusch–Pagan test for homoscedasticity, the Durbin–Watson statistic for residual independence, and Cook’s distance for influential observations.

To account for multiple testing, the Benjamini–Hochberg false discovery rate (FDR) procedure was applied to two families of focal tests: the five academic stress dimensions associated with PMDU and the three associations between PMDU and the psychological outcomes. The association between overall academic stress and PMDU was treated as a single primary test, while gender and age were treated as covariates; these tests were therefore not included in the FDR adjustment. Statistical significance was determined using FDR-adjusted p < .05 for the corresponding families of tests and p < .05 for the single primary test.

### 2.5 Ethical considerations

Ethical approval for this study was granted by the Academic Committee of the School of Humanities, Hunan University of Science and Technology (Approval No. HNUST-RW-ER-2025–003). The study was conducted in accordance with relevant ethical guidelines for research involving human participants. All participants were informed about the study purpose, procedures, potential risks and benefits, confidentiality protections, and their right to withdraw at any time without penalty. Participation was voluntary, and informed consent was obtained prior to data collection. Data were collected via an anonymous questionnaire, de-identified prior to analysis, and stored securely with access restricted to the research team.

## 3 Results

### 3.1 Sample characteristics

Among the participants, 93 were male (18.24%) and 417 were female (81.76%). Participants’ ages ranged from 18 to 30 years (*M* = 19.80, *SD* = 1.47). The sample was predominantly composed of participants aged 18–20 years (18: 23.73%, *n* = 121; 19: 28.43%, *n* = 145; 20: 26.08%, *n* = 133), with smaller proportions aged 21 (9.41%, *n* = 48), 22 (2.35%, *n* = 12), 23 (2.55%, *n* = 13), 24 (2.75%, *n* = 14), 25 (2.35%, *n* = 12), 26 (0.98%, *n* = 5), 27 (0.59%, *n* = 3), 28 (0.39%, *n* = 2), 29 (0.20%, *n* = 1), and 30 (0.20%, *n* = 1).

Participants were recruited from multiple provinces across China. The largest proportions of respondents were from Hunan Province (42.0%, *n* = 214), Jiangsu Province (28.8%, *n* = 147), and Guangdong Province (12.7%, *n* = 65), with additional participants from several other provinces. Although the sample was not nationally representative, these findings indicate a certain degree of geographic diversity within the dataset.

### 3.2 Measurement properties of the PMDU scale

The adapted 15-item PMDU scale demonstrated high internal consistency in the present sample (Cronbach’s α = .922). Corrected item-total correlations ranged from.526 to.762, while inter-item correlations ranged from.218 to.828 (S1 Table in S1 File).

CFA was conducted to examine the factorial structure of the adapted PMDU scale. The single-factor model showed poor fit, whereas the correlated five-factor model demonstrated good fit, χ^2^(80) = 275.38, CFI = .958, TLI = .945, RMSEA = .069, 90% CI [.060,.078], and SRMR = .056. Standardized factor loadings ranged from.624 to.913 (S1 Table in S1 File). The second-order model, in which the five first-order factors loaded on an overarching PMDU factor, also showed acceptable fit, χ^2^(85) = 326.29, CFI = .948, TLI = .936, RMSEA = .075, 90% CI [.066,.083], and SRMR = .066 (Table 2). These findings supported the five-factor structure of the adapted measure, while the acceptable fit of the second-order model provided support for an overarching PMDU construct.

**Table 2:** Confirmatory factor analysis of the adapted PMDU scale.

Model	χ^2^	df	CFI	TLI	RMSEA	90% CI for RMSEA	SRMR
Single-factor model	1400.77	90	.718	.671	.169	[.161,.177]	.108
Correlated five-factor model	275.38	80	.958	.945	.069	[.060,.078]	.056
Second-order five-factor model	326.29	85	.948	.936	.075	[.066,.083]	.066

Note. PMDU = problematic micro-drama use; CFI = comparative fit index; TLI = Tucker–Lewis index; RMSEA = root mean square error of approximation; CI = confidence interval; SRMR = standardized root mean square residual.

Composite reliability values for the five first-order factors ranged from.748 to.926, and AVE values ranged from.537 to.808. The Fornell–Larcker criterion was satisfied for all five factors, and all HTMT values were below.85 (maximum = .775), providing additional evidence of discriminant validity (S2 Table in S1 File).

### 3.3 Descriptive statistics of main variables

Descriptive statistics for the main study variables are presented in Table 3. The mean PMDU score was 44.15 (*SD* = 10.53). Descriptive statistics for academic stress, loneliness, anxiety symptoms, and depressive symptoms are also presented in Table 3.

**Table 3:** Descriptive statistics of main variables.

Variable	Mean/ n	SD/ %	Min	Max	Skewness	Kurtosis
PMDU	44.15	10.53	15	75	−0.22	0.4
AS	81.99	14.99	24	120	−0.16	1.04
Depression	5.9	3.95	0	21	0.7	1.31
Anxiety	6.71	3.84	0	21	0.77	2.16
Loneliness	15.28	5.04	6	30	0.13	0.12

Note. PMDU = problematic micro-drama use; AS = academic stress.

The distributions of the main variables were further examined using skewness and kurtosis statistics. Overall, the values fell within acceptable ranges, suggesting no substantial departures from univariate normality. Specifically, the absolute skewness values were below 1, and the absolute kurtosis values were below 3. Although anxiety symptoms showed the highest kurtosis value (2.16), this value remained within the commonly accepted limits for parametric analyses [43].

### 3.4 Correlation analysis

Pearson correlation analyses were conducted to examine the associations among the main study variables. As shown in Table 4, problematic micro-drama use (PMDU) was positively correlated with academic stress, loneliness, depressive symptoms, and anxiety symptoms (all p < .01). Academic stress was also positively associated with loneliness, depression, and anxiety.

**Table 4:** Correlations among main variables.

Variable	1	2	3	4	5
PMDU	1.0	0.493	0.327	0.312	0.336
AS	0.493	1.0	0.415	0.445	0.397
Depression	0.327	0.415	1.0	0.84	0.673
Anxiety	0.312	0.445	0.84	1.0	0.719
Loneliness	0.336	0.397	0.673	0.719	1.0

Note. All correlations were significant at p < .01. PMDU = Problematic Micro-Drama Use; AS=Academic Stress.

To further examine the relationships among the five dimensions of academic stress, additional correlation analyses were conducted (Table 5). The results showed that coursework burden, academic climate, learning effectiveness, academic competition, and employment prospects were all positively intercorrelated. The correlation coefficients ranged from moderate to relatively strong, indicating positive intercorrelations among the stress dimensions. However, subsequent multicollinearity diagnostics indicated that VIF values remained within acceptable ranges.

**Table 5:** Correlations among academic stress dimensions.

Variable	1	2	3	4	5
CB	1.0	0.583	0.663	0.708	0.518
AC	0.583	1.0	0.674	0.624	0.501
LE	0.663	0.674	1.0	0.78	0.7
CO	0.708	0.624	0.78	1.0	0.636
EP	0.518	0.501	0.7	0.636	1.0

Note. All correlations were significant at *p* < .01. AS = Academic Stress; CB = coursework burden; AC = academic climate; LE = learning effectiveness; CO = academic competition; EP = employment prospects.

### 3.5 Regression analyses

Diagnostic analyses indicated no major violations of the assumptions underlying the regression models. Durbin–Watson statistics ranged from 1.957 to 2.241, indicating no substantial residual autocorrelation. Breusch–Pagan tests were nonsignificant across all models (all *p* > .36), providing no evidence of heteroscedasticity. VIF values were below 4, indicating no problematic multicollinearity, and no observation had a Cook’s distance greater than 1. Residual distributions showed no substantial deviations that would preclude the use of linear regression.

To examine whether academic stress was associated with problematic micro-drama use (PMDU), a multiple linear regression analysis was conducted while controlling for gender and age. As presented in Table 6, academic stress was positively associated with PMDU (*β* = .447, *p* < .001). The overall model was statistically significant (*F* = 55.53, *p* < .001), explaining 24.8% of the variance in PMDU (*R*^2^ = .248). Gender and age were not significantly associated with PMDU in this model.

**Table 6:** Multiple linear regression analyses predicting PMDU and psychological outcomes.

Model 1. Predicting PMDU from Overall Academic Stress						
Predictor	*B*	*SE*	*β*	*t*	*p*	
AS	0.353	0.028	.447	12.682	<.001	
Gender	−1.626	1.095	−.052	−1.484	.138	
Age	−0.281	0.218	−.045	−1.290	.198	
Model statistics	*R*² = .248	Adj.*R*² = .244	*F* = 55.53		<.001	
**Model 2. Predicting PMDU from Five Academic Stress Dimensions**						
Predictor	*B*	*SE*	*β*	*t*	*p*	FDR-adjusted *p*
CB	0.129	0.178	.035	0.724	.469	.474
AC	0.434	0.201	.117	2.160	.031	.078
LE	0.983	0.216	.292	4.544	<.001	<.001
CO	0.135	0.187	.043	0.723	.470	.474
EP	0.102	0.142	.033	0.717	.474	.474
Gender	−1.769	1.095	−.056	−1.616	.107	
Age	−0.317	0.219	−.050	−1.447	.149	
Model statistics	*R*² = .274	Adj.*R*² = .264	*F* = 27.14		<.001	
**Model 3. Predicting Loneliness**						
Predictor	*B*	*SE*	*β*	*t*	*p*	FDR-adjusted *p*
PMDU	0.158	0.020	.331	7.884	<.001	<.001
Gender	−0.018	0.555	−.001	−0.032	.974	
Age	−0.205	0.113	−.076	−1.813	.070	
Model statistics	*R*² = .111	Adj.*R*² = .106	*F* = 21.11		<.001	
**Model 4. Predicting Depressive Symptoms**						
Predictor	*B*	*SE*	*β*	*t*	*p*	FDR-adjusted *p*
PMDU	0.121	0.016	.323	7.670	<.001	<.001
Gender	−0.012	0.438	−.001	−0.027	.978	
Age	−0.091	0.089	−.043	−1.023	.307	
Model statistics	*R*² = .105	Adj.*R*² = .100	*F* = 19.78		<.001	
**Model 5. Predicting Anxiety Symptoms**						
Predictor	*B*	*SE*	*β*	*t*	*p*	FDR-adjusted *p*
PMDU	0.111	0.015	.305	7.196	<.001	<.001
Gender	0.142	0.425	.014	0.333	.739	
Age	−0.200	0.087	−.097	−2.313	.021	
Model statistics	*R*² = .096	Adj.*R*² = .091	*F* = 17.86		<.001	

Note. PMDU = Problematic Micro-Drama Use; AS = Academic Stress; CB = coursework burden; AC = academic climate; LE = learning effectiveness; CO = academic competition; EP = employment prospects.

To further investigate which dimensions of academic stress were associated with PMDU, the five dimensions of academic stress were simultaneously entered into the regression model. Before correction for multiple comparisons, learning effectiveness pressure (*β* = .292, *p* < .001) and academic climate pressure (*β* = .117, *p* = .031) were positively associated with PMDU. After applying the Benjamini–Hochberg FDR correction across the five academic stress dimensions, only learning effectiveness pressure remained statistically significant (FDR-adjusted *p* < .001), whereas the association with academic climate pressure did not remain statistically significant (FDR-adjusted p = .078). Coursework burden, academic competition, and employment prospects were not significantly associated with PMDU. The overall model was statistically significant (*F* = 27.14, p < .001), explaining 27.4% of the variance in PMDU (*R*^2^ = .274).

Three additional regression analyses were conducted to examine whether PMDU was associated with loneliness, depressive symptoms, and anxiety symptoms while controlling for gender and age. PMDU was positively associated with loneliness (β = .331, p < .001), depressive symptoms (β = .323, p < .001), and anxiety symptoms (β = .305, p < .001). All three associations remained statistically significant after Benjamini–Hochberg FDR correction (all FDR-adjusted p < .001). The regression models explained 11.1% of the variance in loneliness (R^2^ = .111), 10.5% of the variance in depressive symptoms (R^2^ = .105), and 9.6% of the variance in anxiety symptoms (R^2^ = .096). Among the covariates, age showed a small negative association with anxiety symptoms (β = −.097, p = .021), whereas gender was not significantly associated with the psychosocial outcomes.

## 4 Discussion

### 4.1 Principal findings

This study examined the associations between academic stress, PMDU, and mental health among Chinese university students. Higher academic stress was associated with greater PMDU, and PMDU was positively associated with loneliness, depressive symptoms, and anxiety symptoms. These findings suggest that problematic micro-drama viewing may be relevant to both academic stress and psychological well-being. Given the cross-sectional design, however, the direction of these associations cannot be determined.

### 4.2 Academic stress dimensions and PMDU

Overall academic stress was positively associated with PMDU (*β* = .447, *p* < .001). Students under greater academic stress may be more likely to turn to digital entertainment for temporary distraction or relief from academic demands [44,45]. Micro-dramas are particularly accessible in this respect because individual episodes are short and can be viewed during brief breaks. Their fast-paced and serialized narratives may also encourage continued viewing. This interpretation is broadly consistent with compensatory media use research, which has linked stress and other negative experiences with increased reliance on digital media [46,47].

The dimension-level analysis provides a more specific picture. Learning effectiveness pressure remained significantly associated with PMDU after the other dimensions were included in the model and after correction for multiple comparisons (*β* = .292, FDR-adjusted *p* < .001). Students who feel that they are not learning effectively may experience frustration, worry, or self-doubt [48–50]. Micro-drama viewing may offer a temporary break from these experiences. However, the present study did not measure students’ motives for viewing, so this explanation requires further investigation.

Academic climate pressure was associated with PMDU before correction for multiple comparisons (*β* = .117, *p* = .031), but the association was no longer statistically significant after FDR adjustment (FDR-adjusted *p* = .078). This result should therefore be interpreted cautiously. Coursework burden, academic competition, and employment prospects were also not significantly associated with PMDU in the multivariable model. All five dimensions were positively correlated with PMDU at the bivariate level, suggesting that the nonsignificant coefficients in the multivariable model may partly reflect overlap among the different forms of academic stress.

### 4.3 PMDU and mental health outcomes

PMDU was positively associated with loneliness (*β* = .331), depressive symptoms (*β* = .323), and anxiety symptoms (*β* = .305), with all three associations remaining significant after correction for multiple comparisons. The regression models explained approximately 10% of the variance in their respective outcomes. The similar pattern across the three outcomes suggests that PMDU is associated with several aspects of psychological distress rather than with a single mental health outcome. However, given the cross-sectional design, these associations should not be interpreted as evidence that PMDU leads to poorer mental health.

Several factors may help explain these associations. Micro-dramas typically use fast-paced storytelling, frequent plot developments, and cliffhanger endings, while video platforms make it easy to continue from one episode to the next. These features may make it difficult for some viewers to stop when they originally intended. Extended viewing may also interfere with sleep, studying, physical activity, or social interaction [21–26], all of which are relevant to psychological well-being. These possible mechanisms were not directly examined in the present study and require further investigation.

PMDU and loneliness. PMDU was positively associated with loneliness (*β* = .331). One possible explanation is that heavy viewing reduces time available for face-to-face interaction with friends, classmates, or family members [51–54]. Drama viewing may also provide a temporary sense of companionship through involvement with characters and stories, but this is not equivalent to interpersonal support [55,56]. At the same time, the relationship may operate in the opposite direction: students who already feel lonely may be more likely to spend time watching narrative content for companionship or distraction [57,58]. Longitudinal research is needed to distinguish between these possibilities.

PMDU and depressive symptoms. PMDU was also positively associated with depressive symptoms (*β* = .323). Problematic viewing may interfere with daily routines by delaying sleep, contributing to procrastination, or reducing time available for academic work [59–61]. When these disruptions accumulate, students may experience greater academic difficulty and negative self-evaluation [62,63]. Sleep disruption and reduced engagement in physical and social activities are also associated with depressive symptoms [64–66]. These factors may help explain the observed relationship, although the present data cannot establish whether problematic viewing precedes depressive symptoms or whether students experiencing depressive symptoms are more likely to engage in problematic viewing.

PMDU and anxiety symptoms. PMDU was positively associated with anxiety symptoms (*β* = .305). One possible link is sleep disruption. Continued viewing, particularly at night, may delay bedtime or reduce sleep quality, both of which are associated with anxiety symptoms [27,67,68]. Anxiety may also contribute to problematic viewing if students use micro-dramas to distract themselves from worry or academic pressure. The direction of this relationship cannot be established from the present cross-sectional data.

### 4.4 Theoretical and practical implications

Theoretically, these findings advance research on stress, digital media use, and student mental health by focusing on PMDU as a distinct, narrative-based form of mobile engagement. Unlike non-serial short-form browsing, micro-dramas combine brief episodes with rapid emotional pacing, serialized storytelling, and cliffhanger-driven continuation, which may make disengagement particularly difficult. Consistent with stress-and-coping and compensatory media use perspectives, the positive association between academic stress and PMDU suggests that problematic viewing may serve as a form of stress-related coping. At the dimensional level, learning effectiveness pressure was the only academic stress dimension that remained significantly associated with PMDU after correction for multiple comparisons. However, general coping frameworks do not fully explain why a particular media format becomes difficult to control. Future models of PMDU may therefore benefit from integrating stress and coping processes with media-specific affordances and psychological need-related mechanisms. The associations between PMDU and loneliness, anxiety, and depressive symptoms further suggest the value of examining multiple dimensions of psychological well-being.

Practically, the findings suggest that universities may benefit from addressing both academic stress and problematic micro-drama viewing. Counseling and student mental health services could help students identify stress-related viewing triggers and develop alternative coping strategies, while academic support services could provide assistance with study planning and perceived learning difficulties. Digital well-being education could also encourage students to monitor and structure their viewing through strategies such as time limits or episode caps. These approaches are intended to strengthen self-regulation and reduce interference with academic routines rather than discourage recreational micro-drama viewing. Platform-level measures, such as break prompts and bedtime reminders, may provide additional support, although their implementation would require collaboration beyond the university setting.

### 4.5 Limitations and future directions

Several limitations should be acknowledged. First, the cross-sectional design precludes conclusions about causality or temporal ordering among academic stress, PMDU, and psychological outcomes. Longitudinal and experimental studies are needed to clarify the direction and potential mechanisms of these associations. Second, all variables were assessed using self-report measures, which may be affected by recall bias, social desirability, and common method variance. Future research could incorporate objective behavioral indicators, such as actual viewing duration or platform-recorded usage.

Third, the sample was substantially imbalanced by gender, with female participants accounting for 81.76% of the sample. This imbalance was not intentional but resulted from the recruitment process. Although gender was included as a covariate in the regression analyses, statistical adjustment cannot fully compensate for an imbalanced convenience sample. Sampling weights were not applied because the study did not use probability-based sampling or a predefined population sampling frame. The findings should therefore not be interpreted as nationally representative of Chinese university students, and future studies should consider more gender-balanced or stratified sampling strategies.

Fourth, the regression models adjusted for gender and age but did not account for several potentially relevant factors, including overall screen time, micro-drama viewing frequency or duration, sleep quality, social support, self-control, and prior mental health status, because these variables were not collected in the survey. Residual confounding therefore cannot be excluded. Future studies should incorporate a broader range of behavioral, psychosocial, and media-use covariates to assess whether the observed associations remain robust after more comprehensive adjustment.

Finally, although the adapted PMDU measure demonstrated high internal consistency and satisfactory factorial validity in the present sample, test-retest reliability could not be evaluated because the cross-sectional survey involved only a single administration of the instrument. In addition, external convergent validity was not directly assessed using an independent related measure. Future studies using repeated assessments and additional validated measures are needed to examine the temporal stability and further establish the validity of the adapted PMDU measure.

## 5 Conclusion

In sum, higher academic stress was associated with greater PMDU among Chinese university students, with learning effectiveness pressure showing a significant dimension-specific association after correction for multiple comparisons. Higher PMDU was also associated with greater loneliness, depressive symptoms, and anxiety symptoms. These findings suggest that PMDU is relevant to both academic stress and psychological well-being among university students and may warrant attention in campus stress-management and digital well-being initiatives.

## Supporting information

S1 File File contains S1 Table and S2 Table. (DOCX)
