# Supplementary material for: Linking academic stress to loneliness, anxiety, and depression: The association of problematic micro-drama use among Chinese university students
Source: PLoS One. 2026 Oct 27;21(10):e0361597. doi: 10.1371/journal.pone.0361597 (PMC13712741; doi:10.1371/journal.pone.0361597)
Supplement: Supplementary file 1 [file pone.0361597.s001.docx]

**Supporting Information: Psychometric Properties of the Adapted PMDU Scale**

**S1 Table and S2 Table**

**S1 Table. Item-Level Psychometric Properties and Inter-Item Correlations of the Adapted PMDU Scale**

**Panel A. Standardized factor loadings and corrected item-total correlations**

| **Factor** | **Item** | **Standardized loading** | **Corrected item-total correlation** |
| --- | --- | --- | --- |
| SCD | PMDU1 | 0.649 | 0.539 |
| SCD | PMDU2 | 0.809 | 0.616 |
| SCD | PMDU3 | 0.827 | 0.635 |
| SCD | PMDU4 | 0.624 | 0.609 |
| AP | PMDU5 | 0.793 | 0.671 |
| AP | PMDU6 | 0.765 | 0.648 |
| AP | PMDU7 | 0.813 | 0.655 |
| ACD | PMDU8 | 0.889 | 0.762 |
| ACD | PMDU9 | 0.913 | 0.716 |
| ACD | PMDU10 | 0.893 | 0.713 |
| IS | PMDU11 | 0.822 | 0.630 |
| IS | PMDU12 | 0.859 | 0.580 |
| IS | PMDU13 | 0.787 | 0.526 |
| IC | PMDU14 | 0.799 | 0.631 |
| IC | PMDU15 | 0.746 | 0.600 |

**Panel B. Inter-item correlation matrix**

| **Item** | **1** | **2** | **3** | **4** | **5** | **6** | **7** | **8** | **9** | **10** | **11** | **12** | **13** | **14** | **15** |
| --- | --- | --- | --- | --- | --- | --- | --- | --- | --- | --- | --- | --- | --- | --- | --- |
| 1 | — |  |  |  |  |  |  |  |  |  |  |  |  |  |  |
| 2 | 0.553 | — |  |  |  |  |  |  |  |  |  |  |  |  |  |
| 3 | 0.504 | 0.703 | — |  |  |  |  |  |  |  |  |  |  |  |  |
| 4 | 0.411 | 0.436 | 0.505 | — |  |  |  |  |  |  |  |  |  |  |  |
| 5 | 0.350 | 0.400 | 0.394 | 0.484 | — |  |  |  |  |  |  |  |  |  |  |
| 6 | 0.347 | 0.393 | 0.355 | 0.389 | 0.622 | — |  |  |  |  |  |  |  |  |  |
| 7 | 0.334 | 0.343 | 0.329 | 0.452 | 0.644 | 0.609 | — |  |  |  |  |  |  |  |  |
| 8 | 0.467 | 0.429 | 0.466 | 0.554 | 0.537 | 0.541 | 0.618 | — |  |  |  |  |  |  |  |
| 9 | 0.408 | 0.375 | 0.418 | 0.512 | 0.515 | 0.515 | 0.583 | 0.810 | — |  |  |  |  |  |  |
| 10 | 0.398 | 0.387 | 0.416 | 0.529 | 0.527 | 0.507 | 0.606 | 0.781 | 0.828 | — |  |  |  |  |  |
| 11 | 0.323 | 0.442 | 0.478 | 0.358 | 0.446 | 0.419 | 0.347 | 0.420 | 0.348 | 0.365 | — |  |  |  |  |
| 12 | 0.243 | 0.444 | 0.428 | 0.319 | 0.379 | 0.366 | 0.308 | 0.375 | 0.343 | 0.333 | 0.705 | — |  |  |  |
| 13 | 0.301 | 0.387 | 0.413 | 0.251 | 0.307 | 0.335 | 0.218 | 0.305 | 0.266 | 0.266 | 0.622 | 0.698 | — |  |  |
| 14 | 0.305 | 0.325 | 0.372 | 0.407 | 0.468 | 0.444 | 0.529 | 0.516 | 0.486 | 0.490 | 0.426 | 0.373 | 0.383 | — |  |
| 15 | 0.337 | 0.373 | 0.385 | 0.319 | 0.406 | 0.432 | 0.404 | 0.489 | 0.485 | 0.429 | 0.416 | 0.364 | 0.409 | 0.596 | — |

**Note.** PMDU = problematic micro-drama use; SCD = social communication difficulties; AP = academic procrastination; ACD = attention concentration difficulties; IS = interpersonal strain; IC = impaired control. Standardized loadings are from the correlated five-factor CFA. Corrected item-total correlations exclude the focal item from the total score. The lower triangle of Panel B reports Pearson inter-item correlations; the diagonal is omitted. Across the 15 items, inter-item correlations ranged from .218 to .828.

**S2 Table. Reliability, Convergent Validity, and Discriminant Validity of the PMDU Factors**

**Panel A. Reliability and convergent validity**

| **Factor** | **Cronbach’s α** | **CR** | **AVE** | **√AVE** |
| --- | --- | --- | --- | --- |
| SCD | 0.811 | 0.820 | 0.537 | 0.733 |
| AP | 0.833 | 0.833 | 0.625 | 0.791 |
| ACD | 0.926 | 0.926 | 0.808 | 0.899 |
| IS | 0.862 | 0.863 | 0.678 | 0.823 |
| IC | 0.747 | 0.748 | 0.598 | 0.773 |

**Panel B. Fornell–Larcker criterion**

| **Factor** | **SCD** | **AP** | **ACD** | **IS** | **IC** |
| --- | --- | --- | --- | --- | --- |
| SCD | 0.733 |  |  |  |  |
| AP | 0.618 | 0.791 |  |  |  |
| ACD | 0.629 | 0.775 | 0.899 |  |  |
| IS | 0.622 | 0.530 | 0.455 | 0.823 |  |
| IC | 0.601 | 0.737 | 0.694 | 0.610 | 0.773 |

**Panel C. Heterotrait–monotrait ratio of correlations (HTMT)**

| **Factor** | **SCD** | **AP** | **ACD** | **IS** | **IC** |
| --- | --- | --- | --- | --- | --- |
| SCD | — |  |  |  |  |
| AP | 0.669 | — |  |  |  |
| ACD | 0.690 | 0.775 | — |  |  |
| IS | 0.618 | 0.535 | 0.455 | — |  |
| IC | 0.635 | 0.732 | 0.696 | 0.623 | — |

**Note.** CR = composite reliability; AVE = average variance extracted; √AVE = square root of AVE. SCD = social communication difficulties; AP = academic procrastination; ACD = attention concentration difficulties; IS = interpersonal strain; IC = impaired control. In Panel B, diagonal values are √AVE and lower-triangle values are latent-factor correlations. The Fornell–Larcker criterion was satisfied for all five factors because each √AVE exceeded the corresponding inter-factor correlations. In Panel C, all HTMT values were below .85 (maximum = .775).
